# Fast Bayesian inference of optical trap stiffness and particle diffusion

**DOI:** 10.1038/srep41638

**Published:** 2017-01-31

**Authors:** Sudipta Bera, Shuvojit Paul, Rajesh Singh, Dipanjan Ghosh, Avijit Kundu, Ayan Banerjee, R. Adhikari

**Affiliations:** 1 Dept of Physical Sciences, Indian Institute of Science Education and Research, Kolkata, Mohanpur 741246, India; 2 The Institute of Mathematical Sciences-HBNI, CIT Campus, Taramani, Chennai 600113, India; 3 Dept of Chemical Engineering, Jadavpur University, Kolkata 700032, India

## Abstract

Bayesian inference provides a principled way of estimating the parameters of a stochastic process that is observed discretely in time. The overdamped Brownian motion of a particle confined in an optical trap is generally modelled by the Ornstein-Uhlenbeck process and can be observed directly in experiment. Here we present Bayesian methods for inferring the parameters of this process, the trap stiffness and the particle diffusion coefficient, that use exact likelihoods and sufficient statistics to arrive at simple expressions for the maximum a posteriori estimates. This obviates the need for Monte Carlo sampling and yields methods that are both fast and accurate. We apply these to experimental data and demonstrate their advantage over commonly used non-Bayesian fitting methods.

Since the seminal contributions of Rayleigh, Einstein, Smoluchowski, Langevin and others^1^, stochastic processes have been used to model physical phenomena in which fluctuations play an essential role. Examples include the Brownian motion of a particle, the fluctuation of current in a resistor, and the radioactive decay of subatomic particles^2^. A central problem is to infer the parameters of the process from partially observed sample paths, for instance, the diffusion constant from a time series of positions, or the resistance from a time series of current measurements, and so on. Bayesian inference provides a principled solution to this inverse problem^3^, making optimal use of the information contained in the partially observed sample path^4^.

The motion of a Brownian particle harmonically trapped in optical tweezers in a volume of a viscous fluid far away from walls is usually modelled by the Ornstein-Uhlenbeck stochastic process^2^,^5^. The stiffness *k* of the harmonic potential, the friction *γ* of the particle, and the temperature *k*_*B*_*T* of the fluid are the three parameters of the stochastic dynamics. For spherical particles Stokes’ law *γ* = 6*πηa* relates the friction to the particle radius *a* and the fluid viscosity *η*, while the Einstein relation^6^, which holds generally, relates the particle diffusion coefficient *D* to the temperature and friction through *D* = *k*_*B*_*Tγ*^−1^. Of these several physical parameters, any *two* may be chosen independently, and it is conventional to choose the ratio *k*/*γ* and *D* to be independent as they relate, respectively, to the mean regression rate *λ* and the volatility *σ* of the Ornstein-Uhlenbeck process (see below).

Reliable estimation of the stiffness is a necessary first step in using tweezers for force measurements. An estimation of the friction, for a particle of known size, provides an indirect measure of the viscosity of the medium. This microscopic method of viscometry is of great utility when sample volumes are in the nanoliter range and conventional viscometric methods cannot be used. Conversely, an estimate of the friction in a fluid of known viscosity provides a method for estimating the particle size. In both these cases, an estimate of the diffusion coefficient provides, by virtue of the Einstein relation, identical information.

Extant protocols for estimating these parameters from discrete observations of the position of the Brownian particle can be divided into “fluctuation” and “response” categories. In the fluctuational methods, the fluctuating position of the particle is recorded and the known forms of the static and dynamic correlation functions are fitted to the data. In response methods, external perturbations are applied to the particle and the known forms of the average response is fitted to the data. Considerable care is needed in these fitting procedures to obtain reliable estimates^7^.

In recent work^8^, Bayesian inference has been applied to the optical tweezer parameter estimation problem. The posterior probability distribution of the stiffness and diffusion coefficient is estimated for a time series of positions, making it a method of the “fluctuation” category. Monte Carlo sampling is needed to compute the posterior distribution and estimation from a time series of 10,000 points requires few tens of seconds. The advantages of the Bayesian method over conventional calibration methods have been discussed at length in this work.

In this paper, we present two Bayesian methods, of the fluctuational category, which do not require Monte Carlo sampling and, consequently, are extremely fast. For example, they estimate the trap stiffness and diffusion coefficient from time series containing a million points in less than a millisecond. The first method extracts information exploiting the Markov property of the sample path and jointly estimates the mean regression rate *k*/*γ* and the diffusion coefficient *D*. The second method extracts information from the equal-time fluctuations of the position, which, in equilibrium, cannot depend on the friction coefficient, and is, then, a function of the stiffness *k* alone. In essence, this is a recasting of the “equipartition” method in the language of Bayesian inference.

The first method, in addition to inheriting the generic advantages of Bayesian inference that have already been pointed out in ref. 8, has several specific advantages. First, it uses the exact expression for the likelihood, which is valid for any Δ*t*, the interval at which the position is observed. Therefore, it works reliably with data acquired at low frequencies. Second, the exact likelihood is expressed in terms of four sufficient statistics, which are quadratic functions of the positions. Their use greatly reduces the computation needed to evaluate the posterior distribution, as four numbers, rather than a large time series now represents the entire information relevant to inference. Finally, we are able to obtain exact maximum a posteriori (MAP) estimates of the mean regression and diffusion coefficients, and their error bars, in terms of the four sufficient statistics. This obviates the need for the Monte Carlo sampling or numerical minimization steps usually required in Bayesian inference. Bayesian credible regions are easily calculated from the analytically obtained error bars. The second method is different from the conventional equipartition method in that it provides a Bayesian error bar, representing a Bayesian credible interval, rather than a frequentist confidence interval^9^. The combined use of exact likelihoods, sufficient statistics and analytical MAP estimates yields both speed and accuracy in parameter estimation.

We apply both methods to experimental data and obtain MAP estimates and error bars that are in excellent agreement with each other. These estimates are found to be in good agreement with the commonly used power spectral density (PSD) calibration method^7^. The Bayesian methods of this paper are implemented in a well-documented, open-source software freely available on GitHub^10^.

The remainder of the paper is organized as follows. In the next section we recall several key properties of the sample paths and distributions of the Ornstein-Uhlenbeck process. We, then, present the Bayesian methods and describe the experimental setup. The Bayesian procedures are then applied to the experimental data. We conclude with a discussion of future directions in the application of Bayesian inference to optical tweezer experiments and advocate its use as a complement to standard non-Bayesian methods.

## Ornstein-Uhlenbeck process

The Langevin equation for a Brownian particle confined in a potential *U* is given by


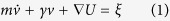


where *ξ*(*t*) is a zero-mean Gaussian white noise with variance 〈*ξ*(*t*)*ξ*(*t*′)〉 = 2*k*_*B*_*Tγδ*(*t* − *t*′) as required by the fluctuation-dissipation theorem. In the limit of vanishing inertia and a harmonic potential, 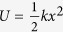, we obtain the overdamped Langevin equation


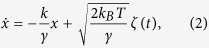


where *ζ*(*t*) is now a zero-mean Gaussian white noise with unit variance. This describes the Ornstein-Uhlenbeck process, whose sample paths obey the Ito stochastic differential equation


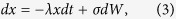


where *λ* is the mean-regression rate, *σ* is the volatility and *W*(*t*) is the Wiener process^5^.

For Brownian motion, the mean regression rate *λ* = *k*/*γ* is the ratio of the stiffness and the friction while the square of the volatility *σ*^2^ = 2*D* is twice the diffusion coefficient *D*. The latter follows by comparing the Langevin and Ito forms of the path equation and recalling the Einstein relation *D* = *k*_*B*_*Tγ*^−1^ between the diffusion and friction coefficients of a Brownian particle. In problems involving Brownian motion, it is convenient to work with the diffusion coefficient, rather than the volatility.

The ratio of *λ* and *D* provides the stiffness


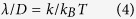


in units of *k*_*B*_*T*. We note that, in general, there is no relation between the mean regression rate and volatility of the Ornstein-Uhlenbeck process and the preceding identity is a consequence of additional *physical* constraints, namely the fluctuation-dissipation and Einstein relations^6^.

The transition probability density *P*_1|1_(*x*′*t*′|*xt*), the probability of a displacement from *x* at time *t* to *x*′ at time *t*′, obeys the Fokker-Planck equation 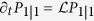, where the Fokker-Planck operator is


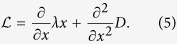


The solution is a normal distribution,


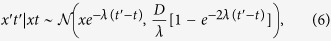


where 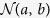 is the univariate normal distribution with mean *a* and variance *b*. This solution is exact and holds for arbitrary values of |*t* − *t*′|. The correlation function decays exponentially,


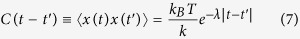


a property guaranteed by Doob’s theorem for any Gauss-Markov process^2^. The Fourier transform of the correlation function gives the power spectral density


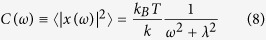


which is Lorentzian in the angular frequency *ω*. The corner frequency *f*_*c*_ = *λ*/2*π* is proportional to the mean regression rate.

The stationary distribution *P*_1_(*x*) obeys the steady state Fokker-Plank equation 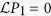 and the solution is, again, a normal distribution,


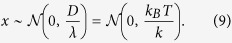


Comparing the forms of *P*_1|1_ and *P*_1_ it is clear that former tends to the latter for |*t* − *t*′| → ∞, as it should.

The transition probability density yields the Bayesian method for jointly estimating *λ* and *D* (and hence *k*), while the stationary distribution yields the Bayesian method for directly estimating *k* alone. We now describe these two methods.

## Bayesian inference

Consider, now, the time series *X* ≡ (*x*_1_, *x*_2_, …, *x*_*N*_) consisting of observations of the sample path *x*(*t*) at the discrete times *t* = *n*Δt with *n* = 1, …, *N*. Then, using the Markov property of the Ornstein-Uhlenbeck process, the probability of the sample path is given by^11^


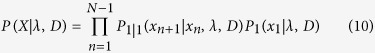


The probability *P*(*λ, D*|*X*) of the parameters, given the sample path, can now be computed using Bayes theorem, as


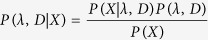


The denominator *P*(*X*) is an unimportant normalization, independent of the parameters, that we henceforth ignore. Since both *k* and *γ* must be positive, for stability and positivity of entropy production respectively, we use informative priors for *λ* and *D, P*(*λ, D*) = *H*(*λ*)*H*(*D*), where *H* is the Heaviside step function. This assigns zero probability weight for negative values of the parameters and equal probability weight for all positive values. The logarithm of the posterior probability, after using the explicit forms of *P*_1|1_ and *P*_1_, is


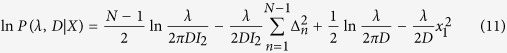


where we have defined the two quantities


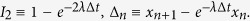


The maximum a posteriori (MAP) estimate (λ^*^, D^*^) solves the stationary conditions ∂ln*P*(*λ, D*|*X*)/∂*λ* = 0 and ∂ln*P*(*λ, D*|*X*)/∂*D* = 0, while the error bars of this estimate are obtained from the Hessian matrix of second derivatives evaluated at the maximum^3^,^12^,^13^. The analytical solution of the stationary conditions, derived in the Supplementary Information, yields the MAP estimate to be


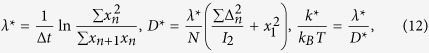


where both *I*_2_ and Δ_*n*_ are now evaluated at *λ* = λ^*^ and the sum runs from *n* = 1, …, *N* − 1. These provide direct estimates of the parameters *without* the need for fitting, minimization, or Monte Carlo sampling.

The error bars are obtained from a Taylor expansion of the log posterior to quadratic order about the MAP value,


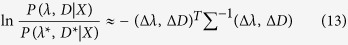


where Δ*λ* = *λ* − λ^*^ and Δ*D* = *D* − D^*^ and ∑^−1^ is the matrix of second derivatives of the log posterior evaluated at the maximum. The elements 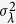, 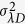, 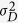 of the covariance matrix ∑ are the Bayesian error bars; they determine the size and shape of the Bayesian credible region around the maximum^13^. Their unwieldy expressions are provided in the Appendix and are made use of when computing credible regions around the MAP estimates. We refer to this Bayesian estimation procedure as “Bayes I” below.

A second Bayesian procedure for directly estimating the trap stiffness results when *X* is interpreted not as a time series but as an exchangeable sequence, or, in physical terms, as repeated independent observations of the stationary distribution *P*_1_(*x*)^12^. In that case, the posterior probability, assuming an informative prior that constrains *k* to positive values, is


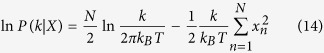


The MAP estimate and its error bar follow straightforwardly from the posterior distribution as


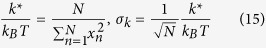


and, not unexpectedly, the standard error decreases as the number of observations increases. This procedure is independent of Δ*t* and is equivalent to the equipartition method when the Heaviside prior is used for *k*. We refer to this procedure as “Bayes II” below.

The posterior probabilities in both methods can be written in terms of four functions of the data


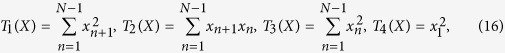


which, therefore, are the sufficient statistics of the problem. The *entire* information in the time series *X* relevant to estimation is contained in these four statistics^12^. Their use reduces computational expense greatly, as only four numbers, rather than the entire time series, is needed for evaluating the posterior distributions.

The posterior distributions obtained above are for flat priors. Other choice of priors are possible. In particular, since both *D* and *k* are scale parameters a non-informative Jeffreys prior is appropriate^3^. Jeffreys has observed, however, that “An accurate statement of the prior probability is not necessary in a pure problem of estimation when the number of observations is large”^3^. The number of observations are in the tens of thousands in time series we study here and the posterior is dominated by the likelihoood rather than the prior. The prior, then, has an insignificant contribution to the posterior.

We note that the error bars obtained in both Bayes I and Bayes II refer to Bayesian credible intervals, which are relevant to the uncertainty in the parameter estimates, given the data set *X*. In contrast, conventional error bars refer to frequentist confidence intervals, which are relevant to the outcomes of hypothetical repetitions of measurement. In general, Bayesian credible intervals and frequentist confidence intervals are not identical and should *not* be compared as they provide answers to separate questions^9^.

A comparison of the estimates for the trap stiffness obtained from these independent procedures provides a check on the validity of the Ornstein-Uhlenbeck process as a data model. Any significant disagreement between the estimates from the two methods signals a breakdown of the applicability of the model and the assumptions implicit in it: overdamped dynamics, constant friction, harmonicity of the potential, and thermal equilibrium. This completes our description of the Bayesian procedures for estimating *λ, D*, and *k*.

## Experimental setup and data acquisition

**Figure 1:**
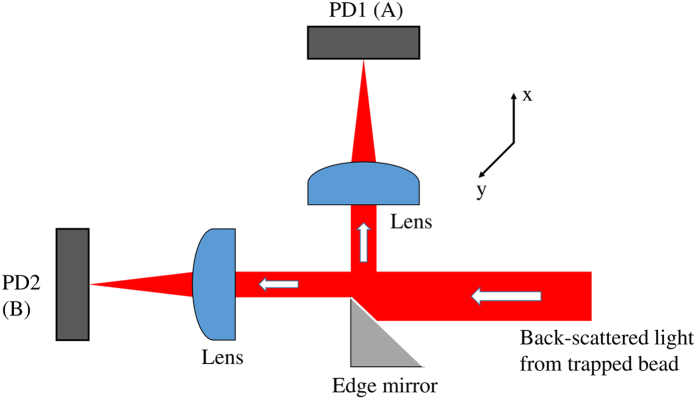
Schematic of balanced detection scheme to measure Brownian motion in the *x* direction from a single trapped polystyrene sphere. Back-scattered light from the trapped sphere is incident on an edge mirror that divides it equally between photodiodes PD1 and PD2, having voltage outputs A and B respectively. The normalized *x* coordinate of the sphere at any instant in time is given by (*A* − *B*)/(*A* + *B*).

We collect position fluctuation data of an optically trapped Brownian particle using a standard optical tweezers setup that is described in detail in ref. 14. Here we provide a brief overview. The optical tweezers system is constructed around a Zeiss inverted microscope (*Axiovert.A1*) with a 100*x* 1.4 numerical aperture (NA) objective lens tightly focusing laser light at 1064 nm from a semiconductor laser (*Lasever*, maximum power 500 mW) into the sample. The back aperture of the objective is slightly overfilled to maximize the trapping intensity. The sample consists of a dilute suspension (volume fraction *ϕ* = 0.01) of polystyrene spheres of diameter 3 *μ*m in 10% NaCl-water solution, around 20 *μl* of which is pipetted on a standard glass cover slip. The total power available at the trapping plane is around 15 mW. A single particle is trapped at an axial distance greater than several times its radius to avoid any wall-effects in the effective drag force due to the water, and it’s motion is observed by back-focal plane interferometry using the back-scattered intensity of a detection laser at 671 nm that co-propagates with the trapping laser. The detection laser power is maintained at much lower levels than that required to trap a particle. The back-scattered signal from the trapped particle is measured using a balanced detection scheme, schematically illustrated in Fig. 1. The back-scattered light beam is incident on an edge mirror which divides it equally into two halves that are focused using two lenses of equal focal length on photodiodes PD1 and PD2 (*Thorlabs* PDA100A-EC Si-photodiodes of bandwidth 2.4 MHz). The voltage outputs *A* and *B*, of PD1 and PD2 respectively, are then combined as (*A* − *B*)/(*A* + *B*) to give the normalized value of the *x* coordinate of motion at any instant of time. The advantage of such balanced detection is that the intensity fluctuations of the laser are present in both beams simultaneously and are thus canceled out when the difference is taken. Note that the direction of the edge mirror decides whether the *x* or *y* coordinate of motion is being measured. The mirror is rotated by 90 degrees to select between the coordinates. The fast response of the photodiodes, with a rise time of 50 ns at highest gain, ensures that spurious correlations are kept to a minimum and the data filtering necessary with slower commercial quadrant photodetectors is avoided entirely. The data from the photodiodes is logged into a computer using a National Instruments DAQ system and Labview at sampling rates between 2–5 kHz. For calibrating the motion, *i.e.* converting the voltage into physical distance which is necessary for measuring the diffusion constant, we employ an acousto-optic modulator that is placed in the back-focal plane of the microscope objective and scan the trapped bead by distances which are determined from the pixel calibration of images taken by the camera attached to the microscope^14^. The balanced detection output is simultaneously measured to yield the voltage-distance calibration of the detection system. The detection signal amplitude for Brownian motion data for 3 *μ*m diameter spheres in water is around 1.5 V/*μ*m and the noise floor is around 5 mV, which implies that we have a sensitivity of around 7 nm (considering signal/noise = 2) for this case. However, since scattering from spheres depends on diameter (generally increasing with diameter) as well as the refractive index of the ambient medium, this value changes when we change spheres or the medium. Typically, the particle localization is within 2–7 nm in our experiments. For the viscosity measurement, we add glycerol to water in fixed proportions to create 5 samples of different viscosity. The viscosity of each sample is then measured by a commercial rheometer (Brookfield DB3TLVCJ0) to match with the experimental results. The voltage-distance calibration is performed every time we change the particle or the ambient medium.

We note that the measured data is a result of a transformation by the detection apparatus of the physical sample paths. The Bayesian modeling of the detection apparatus and the transformations it induces on the physical sample paths is not pursued here. Therefore, we have a problem of pure estimation and there is no attempt to compare between alternative models of the data generation process.

## Results and Discussions

**Figure 2:**
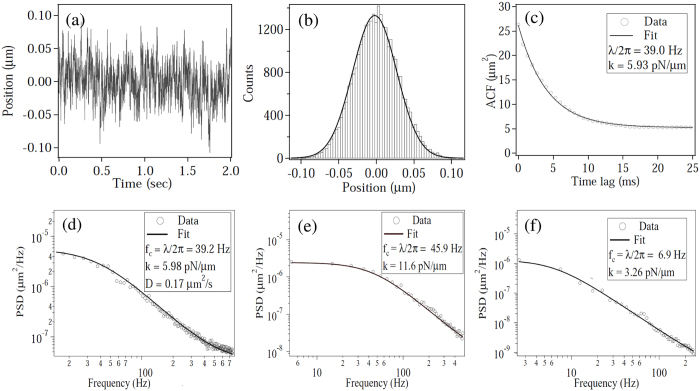
Discrete sample path and empirical statistics of an optically trapped Brownian polystyrene bead of radius a = 3 μm. Panel (**a**) shows discrete observations of one coordinate of the sample path, (**b**) the histogram of the position coordinate, (**c**) the autocorrelation function and (**d**) is the spectral density. Panel (**e**) and (**f**) are the spectral density for 5 and 10 *μ*m diameter polystyrene spheres. The fits of *λ* from the both the autocorrelation and the spectral density depend, respectively, on the number of lags and the number of frequencies used. The guidelines in ref. 7 and 18 are followed in obtaining the fits.

We now present our results. In Fig. (2) we show a typical sample path of one component of motion in the plane of the trap, together with its histogram, autocorrelation function and spectral density. The histogram shows that the distribution of positions is stationary and very well-approximated by a Gaussian. The variance 〈*x*^2^〉 is used in the conventional “equipartition” method to estimate the spring constant *k*, while the fitting of the autocorrelation to the exponential in Eq. (7) or of the spectral density to the Lorentzian in Eq. (8) is used to estimate the spring constant when the friction constant is given. For estimation of the stiffness from the PSD, we employ the procedures suggested in ref. 7, including “blocking” the data with a bin size of 100 points, and setting the frequency range for fitting in order to avoid systematic errors due to reliability issues at both low and high frequencies^7^. However, there exist issues in estimating stiffness from both the equipartition method - where the presence of any additive noise leads to an increase in the variance that leads to over-estimation of the trap stiffness, and the PSD - where the standard systematics related to fitting can be minimized at best but not removed.

**Figure 3:**
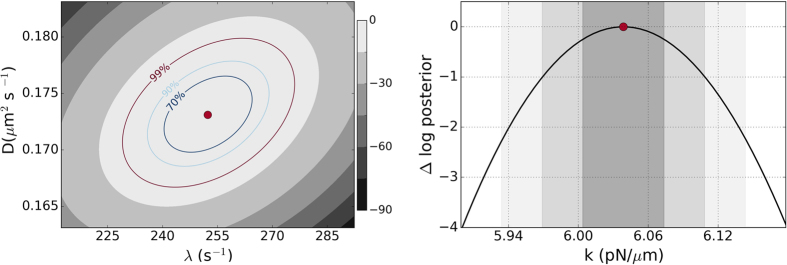
Bayesian posterior probability densities. The left panel shows filled contours of Eq. (11). The MAP estimate, Eq. (12), is marked by the filled dot and contours enclosing 70%, 90% and 99% of the probability are labeled. The middle panel shows Eq. (14). The MAP estimate, Eq. (15), is marked by the filled dot and intervals enclosing 70%, 90% and 99% of the probability are shaded. The two estimates for *k* agree to three decimal places. The analysis is for polystyrene bead of radius *a* = 3 *μ*m.

**Figure 4:**
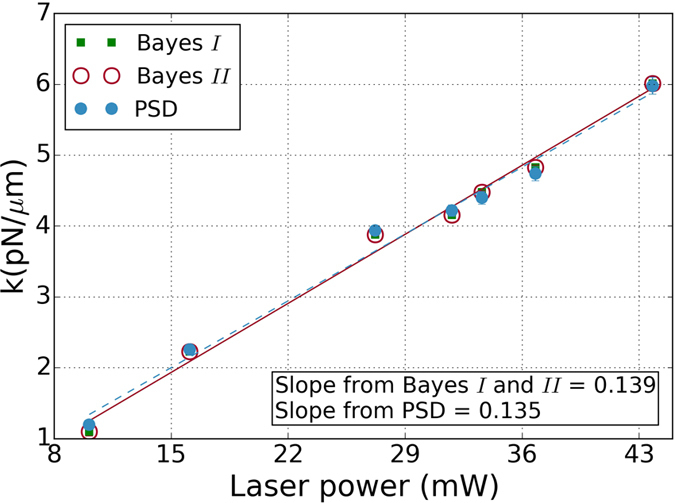
Variation of trap stiffness *k* with laser power estimated by the two Bayesian methods (Bayes I and Bayes II) and by the standard fit to the power spectral density (PSD). The error bars are also shown, but are so small relative to the mean that they are not visible. Solid line is the best fit to Bayes I and II while the dotted line is a best fit to PSD.

The results of Bayesian inference are shown in Fig. (3). In the top panel we show filled contours of the posterior distribution in the *λ* − *D* plane, together with contours of equal probability, for the “Bayes I” method. There is a single maximum at (λ^*^, D^*^) whose numerically computed value is in excellent agreement with the analytical MAP estimates of Eq. (12). In the bottom panel we show the Bayesian posterior distribution for the stiffness for the “Bayes II” method. There is remarkably good agreement between the two Bayesian estimates and the fit to the power spectral density as shown in Fig. (4) and Table. (1). This consistency between three conceptually and procedurally independent methods is evidence for the appropriateness of the data model. The agreement with the spectral density method, shown in the third column of the table, is within 2–3% in all cases, other than the first case where the inherent low stiffness of the trap due to low trapping power led to larger systematics due to the increased influence of the ambient low frequency noise, as we shall discuss later. The typical length of our time series is *N* ∼ 30000 and this gives a Bayesian error bar that is less than 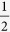
% of the mean. These are well below the systematic errors and the approximately 1.5–2% variability of the estimates obtained from the fitting procedure. The 1*σ* standard errors in the mean are indicated in parenthesis next to each *k* value in Table. (1). Note that for inference of the absolute values of *k* and D^*^, we have used a temperature of 300 K which is the same as the lab environment temperature - this assumption being based on studies in literature^15^ where the effects of laser heating in water has shown to be well below 1 K at the power levels we employ in the trap. However, the fact that the results from Bayes I and Bayes II are extremely close to each other demonstrates that our estimate of temperature is trustworthy. Next, in order to determine the robustness of our experimental techniques, we perform experiments on two other sizes of polystyrene spheres - of diameter 5 and 10 *μ*m, respectively. Representative values of measured stiffness and diffusion constant at a laser power of 43.8 mW are shown in Table 2. It is clear that the remarkable consistency in the values of *k* and D* given by the Bayes I and II methods are preserved, as is the close agreement with the values obtained from the PSD analysis. Note that the stiffness values are not really related to bead size as is well known in literature - the dependence of *k* on bead diameter being rather non-linear^16^. Our next set of measurements are directed towards determining the extent of systematic errors in our measurements. Systematic errors in our experimental apparatus may arise due to various issues including slow drifts of the laser power, beam pointing of the laser, drifts in ambient temperature, coupling with ambient low frequency (typically acoustic) noise sources, and possibly other unidentified reasons. The coupling with ambient low frequency noise sources would manifest themselves at low trapping stiffness, where sudden perturbations could affect the Brownian motion of the bead since the restoring force is less. On the other hand, the affect of slow drifts of experimental parameters would be observed at time series data of longer length. Thus, we attempt to understand the effect of systematics in our experiments using two different approaches: a) by comparing the mean and standard deviation of measured trapping parameters over 3 sets of independent time series data collected at low and high trapping laser powers for the same particle (diameter 3 *μ*m), and b) by comparing the trapping parameters measured on time series data of different length obtained at the same laser power for the same particle. The results are shown in Table 3(a) and (b). In Table 3(a), we observe that at a low laser power of 18.5 mW, we have a standard deviation of around 6.5% in *k* and 4.5% in D^*^, while at the higher power of 43.8 mW, the standard deviation is only around 1.5% for both *k* and D^*^. This demonstrates that the effect of ambient noise does increase at lower trapping powers. In Table 3(b), we show the results of measurements of *k* and D^*^ for time series data of different lengths for the same particle trapped at a laser power of 48 mW (we choose a high laser power since the coupling with the ambient noise is lesser in that case). It is clear that for time series of lengths 5, 10, and 20 s, the variation in the mean of both *k* and D^*^ is only around 1.5%, but there is a large change in the mean values between 7–24% for time series data of 40 and 60 s. In addition, we check that the estimates of *k* and D^*^ over different non-overlapping segments of up to 20 s length in a *single* data set are within the Bayesian error bars for all the time series; however, for 40 and 60 s, the mean values of different non-overlapping sets of 20 s data differ from each other significantly. Thus, it is clear that systematics due to slow drifts in different experimental apparatus occur at time scales longer than 20 s. Note that we perform our experiments on an optical table with active vibration isolation, so that there is no coupling with ambient vibrations, whereas vibrations from the table itself are at much higher frequencies than our region of interest due to the large table mass and are also damped out very fast by the presence of active dampers (we also take care not to place vibrating objects such as power supplies, etc on the table).

To compare the Bayesian estimate for the diffusion coefficient we repeat the experiment for different solvent viscosities keeping both the laser power (corresponding to *k* ~ 6 *pN*) and the particle radius (*a* = 3*μm*) fixed. The Stokes-Einstein relation then provides an estimate of the diffusion coefficient. We compare this estimate with the MAP estimate D^*^ in Table 4 to find agreement to within 10% in all cases. The Stokes-Einstein relation can be used “in reverse” to obtain a MAP estimate of the viscosity, η^*^, which agrees very well with the known viscosity of the mixture. The experiments were performed for five sets of data for each viscosity sample and the mean value of D^*^ has been reported with the corresponding 1*σ* error in parenthesis. The error bars, which are higher than that for the *k* measurement, chiefly reflect the systematic errors in our experimental apparatus that have been described previously and occur at time scales longer than 20*s*, which is the duration over which a single data set is collected. As mentioned earlier, we have checked that the estimates of D^*^over different non-overlapping segments of a *single* data set are within the Bayesian error bars, which again confirms that the errors we observe are due to systematic shifts in the operating conditions of the experiment. The agreement in the viscosity values for that measured in the rheometer and by the Bayesian estimate of D^*^ is within 5% for all cases with the exception of the last, where the enhanced friction caused a shift in *λ* towards lower values, once again increasing the effects of systematics due to ambient low frequency noise sources. Also, since this value corresponded to the highest concentration of glycerol in the water + glycerol mixture, the effects of laser heating could have been more significant^15^, leading to a slight increase of temperature at the trap focal volume which we have not considered in the Bayesian estimate. This “fluctuation” method of estimating the viscosity does not require the application of external fields and is so guaranteed to yield the linear response of the system while the Bayesian analysis extracts, optimally, all information relevant to this estimation problem. The viscosity of nanoliter samples can be estimated by this method, making it an attractive alternative to “response” methods that impose an external shear flow.

## Conclusion

In this work, we have presented an exact Bayesian method for jointly estimating the mean regression rate and the diffusion coefficient of an optically trapped Brownian particle. The trap stiffness in temperature units is obtained as a ratio of the mean regression rate and the diffusion coefficient. We have also rephrased the standard “equipartition” method of directly estimating the trap stiffness as a problem in Bayesian inference. We have assumed that the Ornstein-Uhlenbeck process is the data generating model. More general models, which include the position dependence of the particle friction (as would be the case in the proximity to walls) or the non-Markovian character of the trajectories (as would be the case when momentum diffusion is not slow compared to the time scales of interest) can, with additional effort, be incorporated in the Bayesian framework. Exact analytical solutions will no longer be available and one has to resort to approximations of the likelihood, such as short-time expansions of the Fokker-Planck propagator or numerical solutions of the equivalent stochastic differential equations. These introduce discretization errors which must be carefully evaluated. In contrast, the method presented here is exact and can serve as an useful “null hypothesis” when comparing between different models for the data. In future work, we shall present Bayesian methods for more complex models and provide a fully Bayesian procedure, embodying Ockham’s razor^12^, for the problem of model selection.

Bayesian analysis is generally applicable in studying the dynamics (Brownian or otherwise) of a vast range of mesoscopic particles in diverse trapping environments. While we have focussed on spherical particles here, the method is not restricted thus, and can be applied to non-spherical particles. The rotational and translation motions are typically coupled in such cases, making the analysis more difficult in detail but no different in principle. The capability of modeling the apparatus itself could also be extremely helpful in understanding and improving the experimental techniques employed in optical trapping with the possibility of studying different systematic effects that may influence the trajectory of trapped particles, thus enhancing the capabilities and sphere of influence of optical tweezers.

Bayesian methods for data analysis are not widespread in soft matter, despite of their advantages and demonstrated success in other areas of physics. To quote a popular textbook^17^: “Increasingly, researchers in many branches of science are coming into contact with Bayesian statistics or Bayesian probability theory. By encompassing both inductive and deductive logic, Bayesian analysis can improve model parameter estimates by many orders of magnitude. It provides a simple and unified approach to all data analysis problems, allowing the experimenter to assign probabilities to competing hypotheses of interest, on the basis of the current state of knowledge.” In future, we hope to see many more applications of this “elegant and powerful approach to scientific inference”^17^ to problems in soft matter.

## Additional Information

**How to cite this article**: Bera, S. *et al*. Fast Bayesian inference of optical trap stiffness and particle diffusion. *Sci. Rep.* **7**, 41638; doi: 10.1038/srep41638 (2017).

**Publisher's note:** Springer Nature remains neutral with regard to jurisdictional claims in published maps and institutional affiliations.

## Supplementary Material

Supplemental Information

## Figures and Tables

**Table 1:** Variation of trap stiffness *k* with laser power estimated by the two Bayesian methods of this work (Bayes I and Bayes II) and by the standard fit to the spectral density (PSD)^7^.

Laser power (mW)	*k (pNμm*^−1^)		
	Bayes I	Bayes II	PSD
10.1	1.10 (6)	1.10 (6)	1.20 (2)
16.1	2.23 (1)	2.23 (5)	2.26 (5)
27.2	3.88 (2)	3.88 (5)	3.94 (6)
31.8	4.16 (2)	4.16 (2)	4.22 (8)
33.6	4.48 (2)	4.48 (2)	4.40 (9)
36.8	4.83 (3)	4.83 (2)	4.74 (10)
43.8	6.01 (3)	6.01 (3)	5.98 (12)

The variance of mean is indicated in parentheses. The Bayesian standard error is less than 1% of the mean for each data set.

**Table 2:** Trap stiffness *k* and diffusion constant *D* ^*^ measured for 3, 5, and 10 *μ*m diameter polystyrene spheres trapped at the same laser power.

Particle diameter (*μm*)	*k (pNμm*^−1^)			D^*^ (10^−13^*ms*^−1^)		
	Bayes I	Bayes II	PSD	Bayes I	Bayes II	PSD
3	6.01 (3)	6.01 (3)	5.98 (12)	1.73 (6)	1.74 (6)	1.70 (8)
5	11.5 (1)	11.5 (1)	11.6 (3)	1.015 (10)	1.014 (10)	1.03 (5)
10	3.03 (2)	3.03 (5)	3.26 (6)	0.505 (5)	0.504 (5)	0.50 (1)

The variance of mean is indicated in parentheses.

**Table 3:** Study of systematic error in the experimental apparatus by analysis of particle trajectory for (a) same particle trapped at different laser powers, and (b) time series of different lengths for the same particle trapped at the same laser power.

**(a)**		
**Laser power (mW)**	***k** (**pNμm***^**−1**^)	D^*^ **(10**^**−13**^***ms***^**−1**^)
18.5	2.44 (16)	1.74 (8)
43.8	5.98 (9)	1.75 (3)
**(b)**		
**Time series length (s)**	***k** (**pNμm***^**−1**^)	D^*^ **(10**^**−13**^***ms***^**−1**^)
5	6.77 (6)	1.78 (4)
10	6.58 (5)	1.74 (3)
20	6.72 (4)	1.73 (2)
40	6.29 (2)	2.01 (1)
60	5.10 (1)	1.93 (1)

The variance of mean is indicated in parentheses.

**Table 4:** Bayesian viscometry in an optical trap.

*η*	*D*	D^*^	η^*^
0.00085	1.72	1.73 (6)	0.00084 (3)
0.00089	1.65	1.72 (6)	0.00085 (3)
0.00137	1.07	1.05 (3)	0.00139 (4)
0.00197	0.743	0.732 (11)	0.00200 (3)
0.00243	0.603	0.586 (12)	0.00250 (5)
0.00487	0.301	0.276 (14)	0.00530 (24)

The first column is the viscosity of the solvent as measured in a rheometer and the second column is the diffusion coefficient as given by the Stokes-Einstein relation for that value of the viscosity. The third column is the Bayesian MAP estimate for the diffusion coefficient and the fourth column is the value of the viscosity, as given by the Stokes-Einstein relation for the corresponding value of the diffusion coefficient. There is a good match between the first and fourth columns. Note that the first row is for water while the rest are for water + glycerol samples with increasing glycerol concentration. The variance of mean is indicated in parentheses.
