# Supplementary material for: Fast Bayesian inference of optical trap stiffness and particle diffusion
Source: Sci Rep. 2017 Jan 31;7:41638. doi: 10.1038/srep41638 (PMC5282562; doi:10.1038/srep41638)
Supplement: Supplemental Information [file srep41638-s1.pdf]

Supplemental information: Fast Bayesian inference of optical trap stiffness and particle diffusion

Sudipta Bera,[1] Shuvojit Paul,[1] Rajesh Singh,[2] Dipanjan Ghosh,[3] Avijit Kundu,[1] Ayan Banerjee,[1,*] and R. Adhikari[2,†]

[1] *Dept of Physical Sciences, Indian Institute of Science Education and Research, Kolkata, Mohanpur 741246*
[2] *The Institute of Mathematical Sciences-HBNI, CIT Campus, Taramani, Chennai 600113*
[3] *Dept of Chemical Engineering, Jadavpur University, Kolkata 700032*

(Dated:)

The first partial derivatives of the logarithm of the posterior probability with respect to λ and D are

$$\frac{\partial \ln P}{\partial \lambda} = \frac{N-1}{2}\left(\frac{1}{\lambda} - \frac{I_2'}{I_2}\right) - \frac{\sum \Delta_n^2}{2DI_2} - \frac{\lambda}{2D}\frac{\partial}{\partial \lambda}\left(\frac{\sum \Delta_n^2}{I_2}\right) + \frac{1}{2\lambda} - \frac{x_1^2}{2D}, \qquad \frac{\partial \ln P}{\partial D} = -\frac{N-1}{2D} + \frac{\lambda \sum \Delta_n^2}{2D^2 I_2} - \frac{1}{2D} + \frac{\lambda x_1^2}{2D^2},$$

where $I_2' = 2\Delta t\, e^{-2\lambda\Delta t}$. Setting the second of these equations to zero, D is solved in term of λ and this solution is used in the first equation, together with the large-sample asymptotics

$$\frac{\lambda}{N}\left(\frac{\sum \Delta_n^2}{I_2} + x_1^2\right) \approx \frac{\lambda}{(N-1)}\frac{\sum \Delta_n^2}{I_2},$$

to cancel all D-dependent terms. Setting the resulting equation to zero and solving for λ then yields the MAP estimates in Eq. (12). The second partial derivatives, appearing in Eq.(13), are

$$\Sigma_{11}^{-1} = \frac{\partial^2 \ln P}{\partial \lambda^2} = \frac{N-1}{2}\Big(-\frac{1}{\lambda^2} + \frac{I_2' I_2'}{I_2^2} - \frac{I_2''}{I_2}\Big) - \frac{1}{2\lambda^2} - \frac{1}{D}\frac{\partial}{\partial \lambda}\left(\frac{\sum \Delta_n^2}{I_2}\right) - \frac{\lambda}{2D}\frac{\partial^2}{\partial \lambda^2}\left(\frac{\sum \Delta_n^2}{I_2}\right),$$

$$\Sigma_{12}^{-1} = \frac{\partial^2 \ln P}{\partial D \partial \lambda} = \frac{\sum \Delta_n^2}{2D^2 I_2} + \frac{\lambda}{2D^2}\frac{\partial}{\partial \lambda}\left(\frac{\sum \Delta_n^2}{I_2}\right) + \frac{x_1^2}{2D^2}, \qquad \Sigma_{22}^{-1} = \frac{\partial^2 \ln P}{\partial D^2} = \frac{N-1}{2D^2} - \frac{\lambda}{D^3}\Big(\frac{\sum \Delta_n^2}{I_2} - x_1^2\Big) + \frac{1}{2D^2},$$

where $I_2'' = -4\Delta t^2\, e^{-2\lambda\Delta t}$. All the derivatives are evaluated at the maximum given in Eq.(12). These are assembled into the Hessian matrix $\mathbf{\Sigma}^{-1}$and the matrix is inverted to give the covariance matrix $\mathbf{\Sigma}$ in Eq.(13), whose matrix elements are σ_λ^2, $\sigma_{\lambda D}^2$, σ_D^2

$$\sigma_\lambda^2 = -\frac{1}{\det \mathbf{\Sigma}^{-1}}\Sigma_{22}^{-1}, \qquad \sigma_{\lambda D}^2 = \frac{1}{\det \mathbf{\Sigma}^{-1}}\Sigma_{12}^{-1}, \qquad \sigma_D^2 = -\frac{1}{\det \mathbf{\Sigma}^{-1}}\Sigma_{11}^{-1},$$

where $\det \mathbf{\Sigma}^{-1} = \Sigma_{11}^{-1}\Sigma_{22}^{-1} - \Sigma_{12}^{-1}\Sigma_{21}^{-1}$ and $\Sigma_{21}^{-1} = \Sigma_{12}^{-1}$.

* ayan@iiserkol.ac.in
† rjoy@imsc.res.in
